# Comparative effects of a TLR7/8 agonist and doxorubicin on immune checkpoint modulation and apoptosis in 4T1 breast cancer cells

**DOI:** 10.1016/j.bbrep.2025.102440

**Published:** 2026-01-05

**Authors:** Nazanin Joudaki, Mohsen Tehrani, Abolghasem ajami, Saeid Taghiloo

**Affiliations:** a Department of Immunology, School of Medicine, Mazandaran University of Medical Sciences, Sari, Iran; b Student Research Committee, School of Medicine, Mazandaran University of Medical Sciences, Sari, Iran; c Molecular and Cell Biology Research Center, Mazandaran University of Medical Sciences, Sari, Iran; d Department of Infectious Diseases, Antimicrobial Resistance Research Center, Mazandaran University of Medical Sciences, Sari, Iran; e Thalassemia Research Center, Mazandaran University of Medical Sciences, Sari, Iran

**Keywords:** Breast cancer, TLR 7/8 agonist, R848, Doxorubicin, PVR, PD-L1, Gal-9

## Abstract

**Background:**

Breast cancer is still one of the challenges of medical science, despite fast and remarkable advancements in diagnosis and new therapy approaches. This study aims to explore the individual effects of TLR7/8 agonists and to compare their cytotoxic properties with those of the chemotherapy drug doxorubicin on 4T1 breast cancer cells, as well as to evaluate their role in the modulation of immune checkpoint molecules.

**Methods:**

4T1 cancer cells were treated with the TLR7/8 agonist R848 and doxorubicin for a duration of 48 h. First, the viability of the cells was assessed using the MTT method. The relative expression of mRNA for Gal-9, PD-L1, and PVR was analyzed with HPRT serving as a housekeeping gene. Finally, an apoptosis test was conducted to evaluate the cytotoxic effects of R848 and doxorubicin on 4T1 cells. And the wound healing assay was completed in order to assess the migratory potential of 4T1 cells after treatment with R848 and doxorubicin.

**Results:**

The expression of the PVR and Gal-9 genes in the group treated with R848 showed a decrease compared to the control group, although this change was not statistically significant. In contrast, the expression of PD-L1 and PVR in the group treated with doxorubicin increased significantly when compared to both the control group and the R848 treated group. Additionally, the total apoptosis rate in both the R848 and doxorubicin treatment groups was significantly higher than that in the control group. After 24 h post-scratch, control 4T1 cells showed about 50 % wound closure, while R848 reduced it to 35 %. Doxorubicin lowered migration to under 10 %. By 48 h, control and R848 nearly closed the wound, unlike the DOX group.

**Conclusion:**

R848 and doxorubicin possess anti-proliferative and pro-apoptotic effects on 4T1 cells, doxorubicin effectively induces cell death and boosts immune checkpoint gene expression, while R848 fosters early apoptosis without raising checkpoint ligand expression.

## Highlights

• R848 (TLR7/8 agonist) and doxorubicin decreased the viability of 4T1 cells.
• Doxorubicin greatly elevated the expression of PD-L1 and PVR checkpoint genes.
• R848 induced early apoptosis without increasing immune checkpoint ligands.
• R848 and doxorubicin agents induced apoptosis whereas doxorubicin revealed stronger late-apoptotic activities.

## 1 Introduction

Breast cancer is still one of the challenges of medical science, despite fast and remarkable advancements in diagnosis and new therapy approaches. Breast cancer is a complex disease that encompasses different subtypes. It is particularly prevalent in women, making up nearly one-third of all cancer cases in this population, and has a mortality rate of around 15 % for diagnosed individuals [1,2]. Genetic, environmental, and lifestyle risk factors influence the epidemiology of this disease worldwide [[3], [4], [5]]. Conventional breast cancer treatment strategies include breast cancer ablation therapy, endocrine therapy, chemotherapy, radiation therapy, and immune checkpoint inhibitors. Unfortunately, most of these treatment strategies are associated with a wide range of side effects [1]. In recent years, immunotherapy has developed into a promising method for cancer treatment, employing the body's immune system to seek out and eradicate tumor cells. It has become the standard of care across many cancer types, including lung cancer, melanoma, and others, along with specific patients diagnosed with breast cancer [6,7].

Nonetheless, immunotherapy is not without its challenges. For example, tumor cells can express immune checkpoint modulators like cytotoxic T-lymphocyte-associated protein 4 (CTLA-4) and programmed cell death ligand 1 (PD-L1), which act to suppress the immune response. Also, In breast cancer, malignant cells can circumvent host immune responses via various mechanisms of immune evasion, including the upregulation of immune checkpoint ligands such as programmed death ligand 1 (PD-L1), PD-L2, poliovirus receptor (PVR; CD155), and galectin-9 (Gal-9) [[8], [9], [10]]. Additionally, the composition of microenvironmental cells, such as cancer-associated fibroblasts (CAFs) and cytokine levels, is shaped by the “invasiveness” of breast cancer cells, thus promoting tumor advancement [1].

A viable approach to altering the composition and functionality of tumor immunity in breast cancer involves the inhibition of tumor-promoting inflammatory signaling pathways that are common to both myeloid and lymphoid cells, such as Toll-like receptors (TLRs) [11]. The activation of TLRs not only induces modifications in the activity of myeloid cells within the tumor microenvironment but also amplifies the activity and specificity of adaptive immunity. For instance, recent studies indicate that the activation of TLR7, an endosomal single-stranded RNA (ssRNA) receptor typically linked to viral responses, diminishes PD-1 (programmed cell death protein 1) expression on T cells and boosts the cytotoxic responses of CD8^+^ T cells [12,13].

Additionally, prior research has demonstrated that To TLRs are present in breast cancer cells. In this regard, it was noted that, apart from TLR6, TLR7, and TLR8, the expression levels of the other TLRs were lower in breast cancer tissues compared to normal tissues. Moreover, TLR3 and TLR9 exhibited varying expression levels in estrogen receptors (ER-)/progesterone receptors (PR-) negative breast cancer when contrasted with control tissues [14]. Consequently, TLR-3, TLR-6, TLR7/8, and TLR-9 agonists are considered among the most promising immunotherapeutic agents for breast cancer treatment. TLR agonists produce indirect antitumor effects by stimulating the immune response to inhibit tumor growth and by directly promoting tumor cell proliferation and cytotoxicity [13]. This investigation is designed to examine the direct consequences of TLR 7/8 agonists and to compare them with the chemotherapeutic drug doxorubicin in terms of their cytotoxic effects on breast cancer cells, as well as their role in modulating checkpoint molecules found on the surfaces of these cancerous cells.

## 2 Methods

### 2.1 Chemical compounds and reagents

TLR7/8 agonist R848 (Resiquimod) was purchased from InvivoChem (Illinois, United States). This drug was dissolved in cell culture grade dimethyl sulfoxide (DMSO) (Sigma-Aldrich, Missouri, USA) to a stock concentration of 79.6 mmol/L, stored frozen in aliquots. Doxorubicin hydrochloride (DOX) (10mg/5 ml) was purchased from NanoAlvand manufactures (Adriax10, Iran) as a conventional chemotherapy medication treatment of breast cancer, with a stock concentration of 3.45 mmol/L.

### 2.2 Cell line and culture

The 4T1 murine breast carcinoma cell line was procured from the Biological Resource Center located in Tehran, Iran. These cells were cultured in a complete roswell park memorial institute (RPMI-1640) growth medium (Biowest, Nuaille, France), supplemented with 10 % heat-inactivated fetal bovine serum (Biowest, Nuaille, France), along with 100 units/ml of penicillin and 100 μg/ml of streptomycin (Biowest, Nuaille, France), utilizing 25 cm^2^ culture flasks (SPL Life Sciences, South Korea). The culture flasks were maintained in an incubator set at 37 °C with an atmosphere of 5 % CO_2_ and humidity (Binder, Tuttlingen, Germany).

### 2.3 MTT

To investigate cytotoxicity and determine the optimal dose of TLR agonist and doxorubicin, a 3-(4,5-dimethylthiazol-2-yl)-2,5-diphenyltetrazolium bromide **(**MTT) colorimetric test was employed. In this study, the Sigma MTT kit was used to examine cell viability. First, 8000 cells were seeded in 200 μl of complete dulbecco's modified eagle medium (DMEM) culture medium containing 10 % fetal bovine serum (FBS) in a 96-well plate. They were exposed to increasing concentrations of R848 (3.125, 6.25, 12.5, 25, 50, 100, and 200 μM) and doxorubicin (0.05, 0.1, 0.2, 0.4, 0.8, 1.6, 3.2, and 6.4 μM). After incubation in a 37 °C incubator in the presence of 5 % CO_2_ for 48 h, 20 μl of MTT dye (5 mg/ml) was added to each well and incubated in the dark for 4 h in the incubator. After incubation and observation of black crystals from MTT dye under an inverted microscope, the culture plate was centrifuged at 300 g for 10 min to sediment the crystals. The supernatant was removed, and 100–150 μL of DMSO as a solvent was added to each well and incubated for 20–30 min on a shaker in the dark until the black crystals in the wells were completely dissolved and turned cherry red. Finally, the optical density (OD) of each well was measured at 570 and 720 nm using an ELISA plate reader (Synergy H1 BioTek, Winooski, USA).

### 2.4 RNA extraction and quantitative Real-Time PCR

Total RNA was extracted from 4T1 cells using the Parstous RNA extraction kit (Mashhad, Iran), and then RNA purity was evaluated using a nano-spectrophotometer (WPA, Cambridge, England). Samples with A260/280 ratios of 1.8–2.1 and A260/230 > 1.8 were used for downstream.

Complementary DNA (cDNA) was synthesized through reverse transcription of 1 μg of total RNA within a 20 μl reaction volume composed of 10 μl of 2X Buffer-Mix, 1 μl Ultra-Enzyme Mix, and the requisite RNase/DNase-free water. The reaction mixture was incubated at 25 °C for 10 min, followed by an incubation at 55 °C for 30 min, and the enzymatic activity was terminated by heating at 85 °C for 5 min, employing the Parstous cDNA synthesis kit (Mashhad, Iran). Primer sequences were sourced from the publication by Taghiloo et al. [15] and the primers were acquired from Metabion International AG (Planegg, Germany).

Quantitative Real-Time PCR (qRT-PCR) assays for PD-L1, Galectin-9, PVR, along with the reference gene hypoxanthine-guanine phosphoribosyl transferase (HPRT), were conducted for all experimental groups utilizing the StepOne Real-Time PCR System (Applied Biosystems, California, USA) in conjunction with SYBR green detection dye (Pishtaz-teb, Tehran, Iran). The thermal cycling protocol was established at 1 cycle of 94 °C for 5 min, followed by 40 cycles consisting of 94 °C for 30 s, 60 °C for 30 s, and 72 °C for 30 s. Each experimental run concluded with a melting curve analysis to ensure the absence of primer dimers and to verify amplification specificity. Ultimately, the relative mRNA expression levels of PD-L1, Galectin-9, and PVR were quantified employing the 2^−ΔΔCt^ methodology.

### 2.5 Cell apoptosis examination

4T1 cells were cultured in a 6-well plate for a duration of 48 h at a density of 125,000 cells per well, followed by treatment with R848 at a concentration of 91 μM and doxorubicin at a concentration of 1 μM. Subsequently, the a dual-color FITC-labeled Annexin V/propidium iodide (PI) kit from Parstous (Mashhad, Iran) was utilized to conduct the assay. In accordance with the kit's instructions, the wells were initially washed with phosphate-buffered saline (PBS) and then incubated with 10 % trypsin for 5 min at 37 °C. Once the cells were detached from the plate's surface, they were neutralized using complete medium. The cells were then transferred to a microtube and centrifuged at 300×*g* for 5 min. Following this, 1 ml of phosphate buffer was added to the cells and they were placed at 4 °C for 5 min. The cells were centrifuged again for 5 min at 4 °C and 5000 rpm. The cell pellet was then resuspended in 100 μl of cold binding buffer (1X). To the cell suspension, 1 μl of Annexin V dye was added and incubated for 15 min in the dark. After this incubation, 900 μl of binding buffer (1X) was added to the cell suspension. Subsequently, 1 μl of PI dye was introduced to all groups and incubated for an additional 5 min in the dark. Finally, the cells were analyzed using a Partec PAS flow cytometer system (Partec GmBH, Germany).

### 2.6 Scratch assay for cell migration

The wound healing (scratch) assay was completed in order to assess the migratory potential of 4T1 breast cancer cells after treatment with DOX and R848. 4T1 cells were prepared in 6-well plates at a seeding density of approximately 1 × 10^6^ cells per well and allowed to grow to 90–100 % confluent. Once a monolayer was established, a straight scratch was made through the cell monolayer with a sterile 200 μl pipette tip. All detached cells were gently washed away with PBS and the wells were replaced with medium containing either DOX (1 μM), R848 (91 μM) respectively. Control wells were prepared with medium only, with no drug. Images of the wound area were taken at 0 h (immediately after scratching), 24 h, and 48 h, using inverted microscope.

### 2.7 Statistical analysis

Statistical analyses were conducted using GraphPad Prism 6 (San Diego, CA, USA). The results of the experiment are presented as mean ± standard deviation. To assess the normality of the data distribution, the Kolmogorov-Smirnov and shapiro-wilk test were performed. A one-way analysis of variance (ANOVA) was used to compare the treated and untreated groups, followed by Tukey's post hoc test for multiple comparisons. A significance threshold was set at P values less than 0.05.

## 3 Results

### 3.1 Cell viability of 4T1 breast cancer cells following treatment with R848 and DOX

**Fig. 1:**
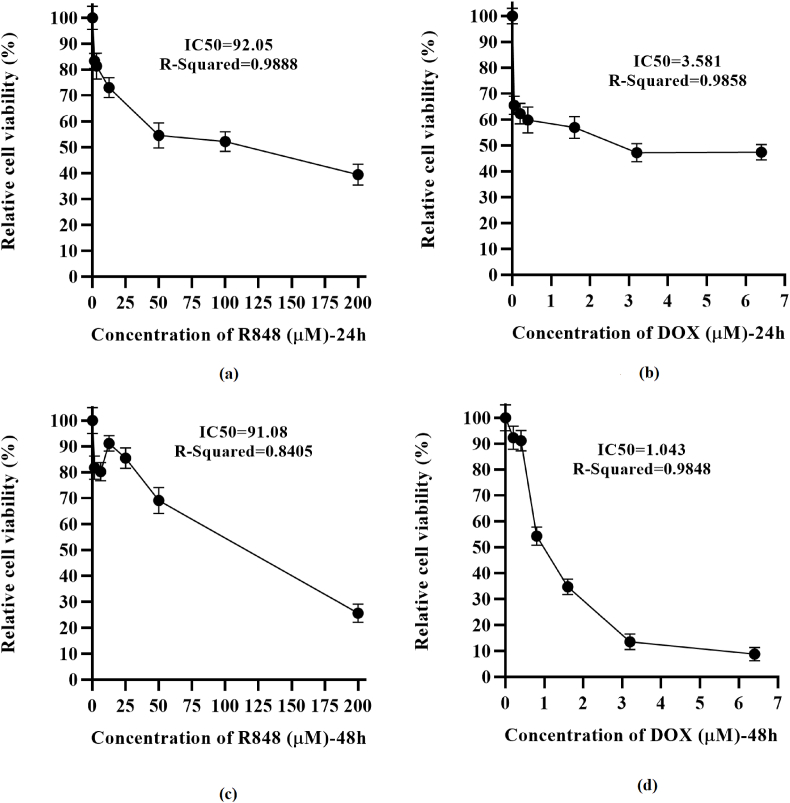
**Determining the IC50 values of R848, and doxorubicin.** 4T1 cells were treated with increasing concentrations of R848 (a) and doxorubicin (DOX) (b) for 24 h, and R848 (c) and DOX (d) for 48 h. Cell viability was determined by measuring the absorbance at 570 nm after the addition of 3-(4,5-dimethylthiazol-2-yl)-2,5-diphenyltetrazolium bromide (MTT) reagent. IC50 value of R848 and DOX were calculated to be 91 μM and 1 μM respectively. Data are presented as mean ± SD from three independent biological replicates (N = 3). Error bars indicate standard deviation.

4T1 cells underwent treatment with progressively higher concentrations of R848 and DOX for durations of 24 and 48 h. The outcomes derived from the cell viability assays revealed that the 48-h treatment demonstrated superior efficacy compared to the 24-h treatment, which we selected for subsequent experimental procedures. Our results indicated that 4T1 cells exhibited a dose-dependent reduction in viability following individual treatments with increasing concentrations of R848 and DOX, resulting in IC50 values of 1 μM and 91 μM, respectively (Fig. 1).

### 3.2 The expression of immune checkpoint ligands on 4T1 breast cancer cells following treatment with R848 and DOX

**Fig. 2:**
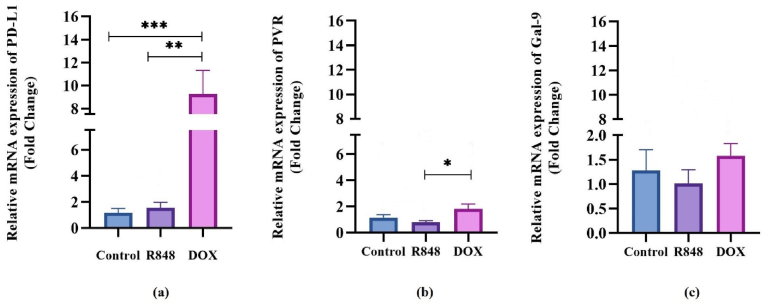
**Effects of R848, and doxorubicin on the expression of PD-L1, Galectin-9, and PVR.** 4T1 cells were incubated with R848 and doxorubicin (DOX) for 48 h. Real-Time PCR was done with specific primers for PD-L1, Galectin-9, PVR, and Hypoxanthine Guanine Phosphoribosyl transferase (HPRT). Relative transcript levels of PD-L1 (a) PVR (b) and Galectin-9 (c) are shown. One-way analysis of variance (ANOVA) with Tukey's post hoc test was used for analyses. The results are presented as mean ± SD. ∗P < 0.05; ∗∗P < 0.01; ∗∗∗P < 0.001; ∗∗∗∗P < 0.0001.

To gain deeper insights into the immune evasion mechanisms driven by TLR stimulation in 4T1 breast cancer cells, we conducted a comprehensive evaluation of the gene expression of key immune checkpoint molecules, including PD-L1, Gal-9, and PVR. This assessment was performed following treatment with DOX and a TLR7/8 agonist R848. We utilized a qRT-PCR assay to quantify the relative expression levels of these molecules, with HPRT designated as the housekeeping internal control. Our findings aim to shed light on the intricate interactions between cancer cells and the immune system, highlighting potential avenues for therapeutic intervention. Our study revealed that the PD-L1 gene expression level was significantly heightened in a set of 4T1 cancer cells subjected to Dox treatment, as opposed to 4T1 cells treated with R848 and the control group, with P-values of <0.01 and < 0.001, respectively (Fig. 2a). The expression level of the PVR gene exhibited a significant difference solely between the two cell groups treated with Dox and R848, yielding P-values of less than 0.05. Nevertheless, the group that received R848 demonstrated a reduction in the expression level of this gene in comparison to the control group, albeit this finding was not statistically significant (Fig. 2b). Also, the Gal-9 gene's expression level did not exhibit significant variation among the three groups; however, treatment with Dox enhanced the expression level, while R848 diminished it relative to non-treated cells (Fig. 2c).

### 3.3 Assessment of apoptosis in 4T1 breast cancer cells following R848 and DOX treatment

**Fig. 3:**
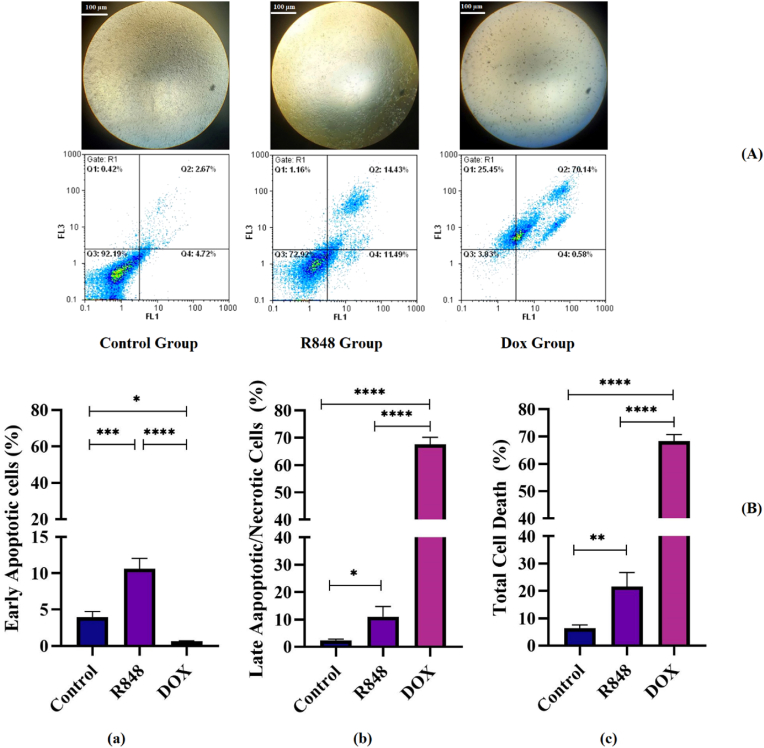
**Effects of R848, and doxorubicin on the apoptosis of 4T1 cells. (A)** 4T1 cells were incubated with R848 and doxorubicin (DOX) for 48 h. Images related to each group are shown under light microscopy, along with the gating strategy used. **(B)** Early Apoptotic Cells (a), Late Aapoptotic/Necrotic Cells (b) and Total Cell Death (c) are shown. One-way analysis of variance (ANOVA) with Tukey's post hoc test was used for analyses. The results are presented as mean ± SD. ∗P < 0.05; ∗∗P < 0.01; ∗∗∗P < 0.001; ∗∗∗∗P < 0.0001. Representative microscopic images of the samples acquired using an inverted microscope with a 4 × objective. Scale bar = 100 μm.

To investigate the role of R848 in cell death and to compare this small molecule with the chemotherapeutic drug DOX, an apoptosis assay was performed. Images related to each group are shown under light microscopy, along with the gating strategy used) Fig. 3A). The results from this assay revealed that R848 significantly augmented early apoptosis when compared to a group of cells treated with DOX and a group of untreated cells, with P-values of <0.0001 and < 0.001, respectively) Fig. 3B–a). The late apoptosis/necrosis parameter was also assessed among these three groups, showing that DOX significantly increased this parameter relative to the control group, with P-values of <0.0001, while R848 also raised this parameter compared to the control group, with P-values of <0.05) Fig. 3B–b). Similarly, the total cell death parameter was significantly elevated in the two groups of cells treated with DOX and R848, with P-values of <0.0001 and < 0.01, respectively, when compared to the control group (Fig. 3B and c).

### 3.4 Assessment of migration in 4T1 breast cancer cells following R848 and DOX treatment

**Fig. 4:**
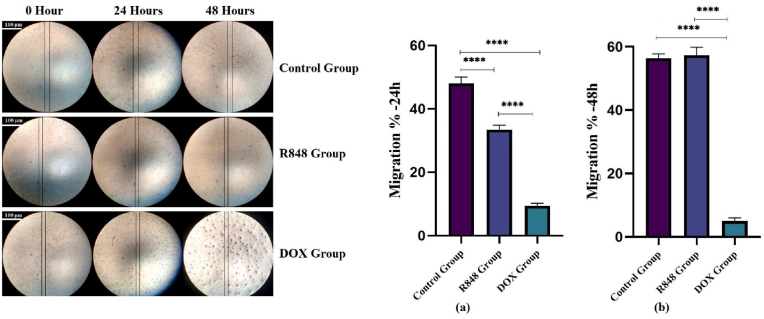
**Effects of R848, and doxorubicin on the migration of 4T1 cells**. Bar graphs represent the percentage of wound closure at 24 h (a) and 48 h (b) in control, R848-treated, and doxorubicin **(**DOX)-treated groups. Data are presented as mean ± SD (n = 3). One-way analysis of variance (ANOVA) with Tukey's post hoc test was used for analyses. The results are presented as mean ± SD. ∗P < 0.05; ∗∗P < 0.01; ∗∗∗P < 0.001; ∗∗∗∗P < 0.0001. Representative microscopic images of the samples acquired using an inverted microscope with a 4 × objective. Scale bar = 100 μm.

The scratch test was conducted to examine the impact of R848 and to compare it with the effect of DOX on the migration of 4T1 cancer cells. To prevent the potential influence of apoptosis on the cell migration assay, the therapeutic dose and duration of drug exposure were selected to minimize significant cytotoxicity, as determined by the viability assay (MTT). Consequently, it is improbable that the reduced migration was a result of apoptosis. At 24 h post-scratch, the control 4T1 cells exhibited a prominent migratory capacity with nearly 50 % wound closure. Treatment with the TLR7/8 agonist R848 significantly decreased cell migration to approximately 35 % compared to the control (p < 0.0001). Doxorubicin demonstrated the strongest inhibitory effect, reducing migration to less than 10 %, which was significantly lower than both the control and R848 groups (p < 0.0001) (Fig. 4a). By 48 h, both the control and R848-treated cells displayed nearly complete wound closure, with no significant difference noted between these two groups. In contrast, the DOX-treated group maintained a markedly reduced migration rate (∼5–8 %), remaining significantly lower than both the control and R848 groups (p < 0.0001) (Fig. 4b).

## 4 Discussion

The immune system of breast cancer patients becomes disrupted during the progression of the disease. Tumor cells utilize various mechanisms to evade immune system surveillance, including the expression of checkpoint proteins such as PD-L1, CTLA-4, Galectin-9, and PVR [16,17]. Additionally, tumor cells can induce exhaustion in immune cells, particularly T cells, which exacerbates disease progression and diminishes patient survival rates [18,19]. Therefore, it is crucial to develop strategies that reduce the expression of these checkpoint molecules while simultaneously promoting tumor cell death and enhancing immune system function. In this context, the use of TLR agonists for treating various cancers, including colon cancer, melanoma [20], leukemia [21], and breast cancer [22], has recently been explored both as a standalone therapy and in combination with other treatments [23]. This study was designed to evaluate the individual effects of doxorubicin and R848 on murine 4T1 breast cancer cells rather than their combined activity. Although combination therapy may provide additional insights, assessing potential synergy or interaction was beyond the scope of the current work and should be explored in future studies. As our data indicate, both medications significantly reduce cell viability in a dose-dependent manner, with IC_50_ values of approximately 1 μM for DOX and 91 μM for R848 at 48 h. This indicates that DOX is a more cytotoxic medication than R848, although the latter also displays significant anti-proliferative activity.

Aside from influencing cell proliferation, our qRT-PCR also revealed changes in the expression of immune checkpoint genes. DOX treatment led to the highly significant upregulation of PD-L1 compared to the control and R848 (p < 0.01 and p < 0.001, respectively). However, previous evidence shows that TLR7/8 activation can modulate downstream nuclear factor kappa-light-chain-enhancer of activated B cells (NF-κB) and Interferon regulatory factor (IRF) pathways, which in turn influence PD-L1 transcription [20]. Another study demonstrated that the nano-formulation of R848 resulted in increased expression of PD-L1 on 4T1 cells. [21]. DOX also led to the significant elevation of PVR levels compared to R848 (p < 0.05). R848 had no significant influence on the expression of PVR but led to a slight reduction in the expression of Gal-9, which remained relatively constant across all groups. DOX did lead to a slight elevation in the levels of Gal-9, while R848 led to a modest reduction. These findings reveal that while DOX is cytotoxic, it can paradoxically facilitate immune evasion by the upregulation of checkpoint ligands. Apoptosis assays also revealed that R848 significantly induced early apoptosis compared to the control and DOX-treated cells (p < 0.0001 and p < 0.001, respectively). Both compounds heightened late and total apoptosis, with DOX exerting a very potent effect (late and total apoptosis p < 0.0001), whereas R848 had significant but less potent effects (p < 0.05 for late and p < 0.01 for total).

In line with our research, DOX-driven PD-L1 upregulation has been reported across a number of cancer models. For instance, in the setting of breast cancer, DOX and stress-inducing insults can increase PD-L1 expression as part of a cell-stress survival program, which can promote immune escape, although such upregulation might not necessarily impart acquired drug resistance [22].

This is also supported by other reports that describe that some chemotherapies, such as DOX, increase PD-L1 surface expression [23,24]. There are some contradictory results, however, where DOX was found to downregulate PD-L1 through processes like TTP-mediated mRNA destabilization or decrease expression on the surface of breast cancer cells while promoting nuclear localization [[25], [26], [27]]. Our finding of PD-L1 upregulation supports the paradigm of stress-induced mechanism. In the case of R848, as a TLR7/8 agonist, it is clearly established as an immune stimulant with recognized anti-tumor activity [28]. R848 is demonstrated to cause apoptosis of tumor cells, reduce tumor vasculature, and promote high mobility group box 1 (HMGB1) release features of immunogenic cell death for example, in non-breast models. It also reshapes tumor immunity, with greater infiltration of effector immune cells and augmented anti-tumor host responses [12,29]. Interestingly, PVR (CD155) is also being increasingly noted with PD-L1 as a checkpoint molecule affecting responsiveness to immunotherapy: high expression of PVR, particularly when co-expressed with PD-L1, is predictive of poor response to PD-1 blockade [30]. In our studies, DOX upregulated both PD-L1 and PVR, stressing a dual-checkpoint upregulation that may lower therapeutic effectiveness unless thwarted by immunotherapy. Our results propose a binary model: although DOX is efficient at killing cancer cells, it also activates cell-intrinsic stress pathways that enhance the expression of immune checkpoint molecules, which can result in a more immunosuppressive phenotype. This aligns with the idea of drug-induced PD-L1 as a pro-survival mechanism [22,26]. Conversely, R848, while less cytotoxic, preferentially causes early apoptosis without promoting checkpoint expression to the same extent. This indicates that R848's immunomodulatory mechanism may involve innate immune pathway activation that does not engage robust survival signaling through checkpoints.

Our data showed that the motility of 4T1 breast cancer cells was highly inhibited by R848 at 24 h, but the inhibition efficacy fell by 48 h. Time-course phenomenon suggests that R848 exhibits transient suppression of the capability of the cells to migrate, possibly due to the temporary modulation of TLR7/8 signaling molecules and cytokine production [31]. Deterioration at subsequent time points might reflect desensitizing of the signaling molecules or compensatory action of pro-migratory mechanisms. In comparison, doxorubicin also significantly suppressed migration at both 24 and 48 h, which is also in line with the known cytotoxic and anti-proliferative activities of doxorubicin [32]. As opposed to doxorubicin that exerts action by DNA damage and apoptosis, R848 exerts action by indirect and transitory immune-modulatory mechanisms. In brief, these data demonstrate that single administration of R848 may not offer long-lasting inhibition of metastasis, but the temporary anti-migratory action of R848 could be utilized along with traditional chemotherapeutic agents like doxorubicin to achieve superior efficacy against breast cancer. Nevertheless, this study has its limitations. It was conducted exclusively in vitro and does not account for the complexity of immune cell interactions and tumor microenvironment dynamics. In addition, we only examined gene transcription, without confirmation of the relevant protein levels or PD-L1, PVR, and Gal-9 functionality. The underlying mechanisms, such as signaling pathways regulating the heterogeneous expression of checkpoints or apoptosis induction, remain elusive.

## 5 Conclusion

In conclusion, this work illustrates that R848 and DOX have anti-proliferative and pro-apoptotic effects against 4T1 cells, though through different molecular signatures: DOX strongly induces cell death but also increases immune checkpoint gene expression, whereas R848 promotes early apoptosis without increasing checkpoint ligand expression. These findings highlight the potential of pairing cytotoxic and immunomodulatory drugs and provide the rationale for subsequent in vivo and mechanistic studies.

## Ethical approval

This study was found to be ethically acceptable by the Ethical Committee of Mazandaran University of Medical Sciences (**IR.MAZUMS.AEC.1403.072**).

## Funding

This work is based upon research funded by 10.13039/501100003968Iran National Science Foundation (INSF), Iran under project NO.4039055 and was supported by 10.13039/501100004160Mazandaran University of Medical Sciences, Iran (Project number: (20824).

## Declaration of competing interest

The authors declare that they have no conflict of interests concerning the contents of this article.

## Data Availability

Data will be made available on request.
